# The Association between Neuroticism and Heart Rate Variability Is Not Fully Explained by Cardiovascular Disease and Depression

**DOI:** 10.1371/journal.pone.0125882

**Published:** 2015-05-07

**Authors:** Iva Čukić, Timothy C. Bates

**Affiliations:** Nagoya University, JAPAN

## Abstract

Neuroticism is associated with cardiovascular disease, autonomic reactivity, and depression. Here we address the extent to which neuroticism accounts for the excess heart disease risk associated with depression and test whether cardiac autonomic tone plays a role as mediator. Subjects were derived from a nationally representative sample (n = 1,255: mean age 54.5, *SD* = 11.5). Higher neuroticism was associated with reduced heart rate variability equally under rest and stress. The baseline structural equation model revealed significant paths from neuroticism to heart rate variability, cardiovascular disease and depression, and between depression and cardiovascular disease, controlling for age, sex, height, weight, and BMI. Dropping both the neuroticism to heart rate variability, and neuroticism to heart disease paths significantly reduced the model fit (*p* < .001 in each case). We conclude that neuroticism has independent associations with both autonomic reactivity and cardiovascular disease, over and above its associations with depression and other related variables.

## Introduction

Cardiovascular disease (CVD) and depression are two of the major disease burdens [1,2]. The two diseases are comorbid [3,4], and some research suggests they may causally influence each other [5–7]. However, a third variable, the personality trait of neuroticism, is also strong and well-documented risk factor for depression [8,9], and, to a lesser degree, for CVD onset [10,11] and mortality [12,13] (although some studies failed to detect the latter link: see, for example [14]). This raises the possibility that neuroticism may account, in part, for the excess heart disease risk associated with depression. One suggestion for understanding these relations is to incorporate measures of autonomic tone, a function strongly linked to negative affect [15]. Here we investigate this possibility using heart rate variability (HRV) a normally varying biomarker based on beat to beat intervals [16,17] and previously linked to CVD and depression [18,19].

Reduced HRV has been associated with elevated levels of CVD [20–23], and depression [24,25]. A causal link between heart function and depression was recently demonstrated in a sample of over million Swedish male conscripts [26]. Tulen et al. [27] report that reduced HRV in major depressive disorder may be dependent on high levels of trait anxiety, potentially supporting a chronic link of HRV to neuroticism rather than an acute link to depressed mood. Here we test whether neuroticism and HRV are associated controlling for their mutual associations with depression and cardiovascular disease, and comorbidity between the two diseases.

**Fig 1:**
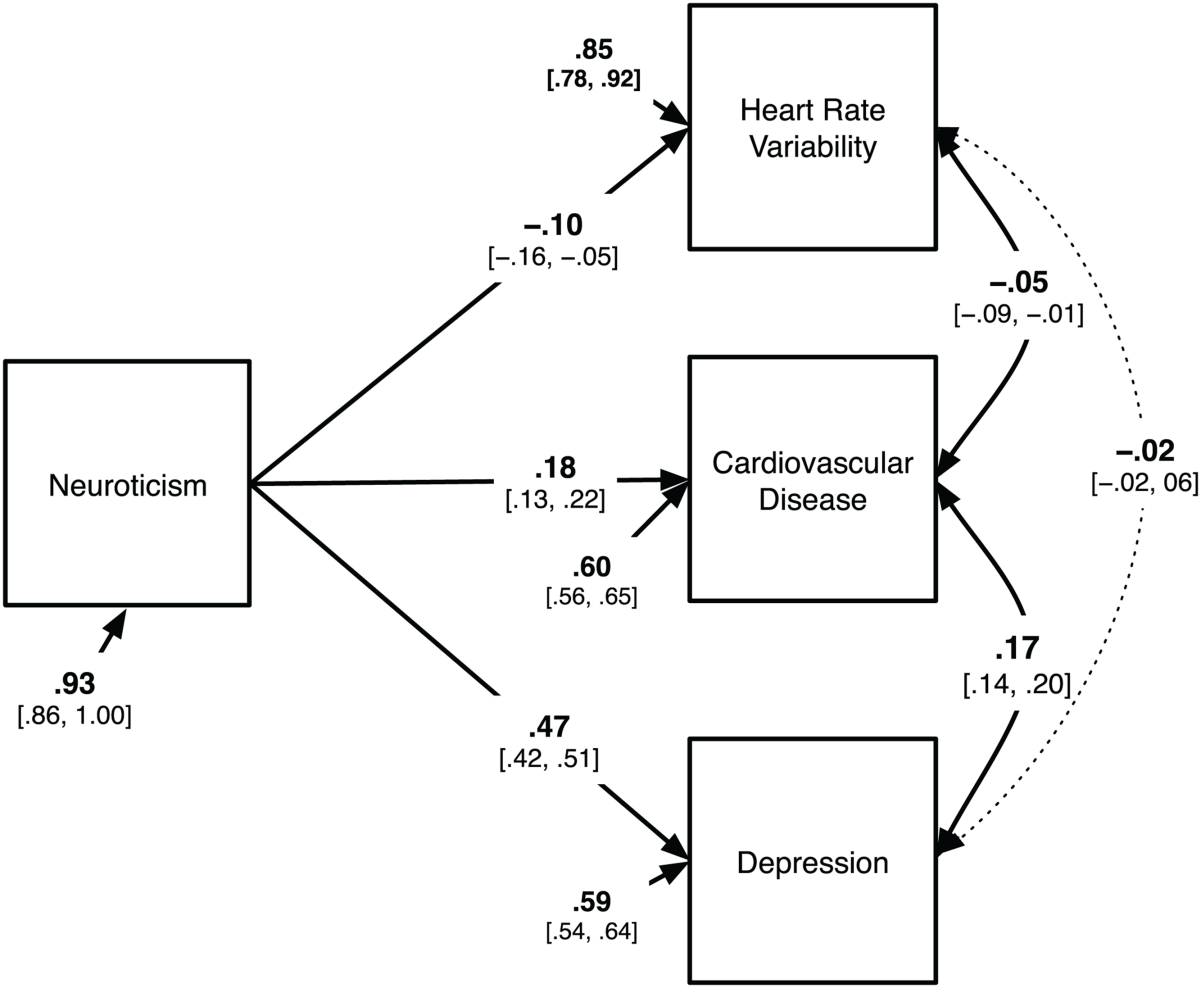
Structural model of the relationship of neuroticism with HRV, cardiovascular disease, and depression. Dotted paths are non-significant (95% CIs in brackets). Note. Covariates (age, sex, height, weight, BMI) are omitted for clarity. See S1 Table for the full list of path estimates.

We tested this hypothesis using path structural equation modelling (SEM) in a large population-based sample. This allows explicit testing of paths in the presence of mediating variables and other associations among variables [28]. Our baseline model is shown in Fig 1. We tested modifications of this model to examine support for direct influences of neuroticism on HRV, and for indirect influences via cardiovascular disease or depression. A significant reduction in model fit for a model lacking a direct contribution of neuroticism to HRV would support the hypothesis that associations of neuroticism with HRV cannot be explained by links to CVD and/or depression alone.

We were able to examine HRV measured in two conditions: at rest, and under stress. This allowed initial tests of whether neuroticism functions as a trait marker for cardiovascular health (i.e., is associated with HRV under both baseline and stress conditions, our preferred hypothesis) or was rather related to HRV only in the stress condition. We first tested this relationship, and then moved to the main modeling hypotheses: that neuroticism would show significant path loadings on heart disease, on depression, and on HRV, independent of each other. All analyses controlled for covariates linked to HRV: age, sex, height, weight, and BMI.

## Method

### Participants

Participants were members of the Project 4: Bioindicators of the MacArthur Foundation Survey for Midlife Development in the U.S cohort (MIDUS) follow-up study (MIDUS II). MIDUS I was a nationally representative sample of 7,108 respondents derived from 50,000 households selected by random telephone dialing [29]. All MIDUS I participants were followed-up in the MIDUS II National Survey. These participants were assessed on a wide range of variables, including demographic characteristics, personality, and extensive mental and physical health measures during a 30-minute phone interview. Of these 5,895 participants, 1,255 were selected to participate in the Bioindicators project (Project 4), designed to add comprehensive biological assessments on a subsample of MIDUS respondents. Project 4 participants were selected based on having previously participated in either the longitudinal survey sample (n = 1,054) or Milwaukee sample (n = 201). This subsample attended a two-day hospital clinic visit where they underwent a series of neuroendocrine, cardiovascular, immune and physical assessments, including the continuous ECG recording reported here. A detailed description of the Project 4 sample composition is provided elsewhere [30]. All available data from these 1,255 participants in Project 4 were included in the present analyses (mean age 54.52 years, *SD* = 11.71). In total, there were 542 males (mean age 55.14 years, *SD* = 11.93) and 713 females (mean age 54.05 years, *SD* = 11.53).

### Measures

#### Neuroticism

Neuroticism was assessed using an adjective-rating scale derived from existing Big Five trait lists and inventories [31]. The adjectives were: “moody”, “worrying”, “nervous” and “calm”. Participants rated themselves on each item using a Likert scale ranging from 1: “Not at all” to 4: “A lot”. The scale is a valid and reliable assessment of neuroticism [32], and correlates highly with standard neuroticism scales [33].

#### Depression

The clinical depression measure was part of the short form of the Composite International Diagnostic Interview, Version 10 (CIDI) [34]. This screening interview is based on the definitions and criteria as described by the third edition-revised of the American Psychiatric Association’s (APA) Diagnostic and Statistical Manual of Mental Disorders (DSM III-R) [34]. For the diagnosis of major depression, a period of at least two weeks of either depressed affect or anhedonia felt most of the days was required over the 12 months prior to the interview, with at least of four other associated symptoms including disrupted eating or sleeping habits, problems with concentration, or suicidal thoughts or actions. The final variable ranged from 0 to 7, where 0 represented a participants diagnosed as negative for major depression, and scores between one and seven represented the range of the symptoms severity [35]. The scale showed satisfactory validity and reliability [36].

#### Heart Disease

Heart disease was assessed via a pre-screening question: “*Have you ever had any of the following conditions/illnesses*?*—Heart disease*”. If the subject did not respond “no”, they were asked if they had heart disease diagnosed by a physician, and this binary variable was used here [29].

#### HRV measures

Continuous recording of the electrocardiogram (ECG) was taken as part of Project 4 of Wave II of the MIDUS study, conducted 2005–2009. The recording was made in a seated position, during the psychophysiology protocol that consisted of a baseline period followed by a cognitive stressor, recovery period, and the second cognitive stressor in the duration of six minutes each. The beat-to-beat waveforms were used to assess heart rate variability, namely, variability of intervals between two consecutive ventricular depolarizations (also known as RR, or NN intervals). Analog signals were digitalized at a sampling rate of 500 Hz by a 16-bit National Instruments analog-to-digital (A/D) board, and then processed by proprietary event detection software that identified RR waves. Researchers then visually inspected waveforms to correct any software errors, following the standard procedures [37,38]. The resulting waveforms were then used to calculate standard measures of cardiovascular reactivity from both time and frequency domain. Standard deviation of beat-to-beat intervals (SDRR) was used as a measure of general autonomic nervous system function variability [39]. Frequency domain measures were used to assess sympathetic and vagal activity separately, namely low frequency power (LF: 0.04–0.15 Hz) for the former; and high frequency (HF: 0.15–0.50 Hz) to assess the latter [39,40]. These measures proved to be reproducible and with useful prognostic value in both long term and short term recordings in clinical patients as well as in normal subjects [39]. All HRV measures were log-transformed.

#### Cognitive stressors

HRV was measured under two conditions: Stress and Baseline. Stress was induced using two cognitive stressors: mental arithmetic and attentional control (Stroop). The MATH task [41,42] is a standard mental arithmetic stressor used in laboratory studies of heart reactivity. Subjects perform addition and subtraction tasks that are adaptively altered to increase or decrease difficulty as a stressor. The second cognitive stressor, the Stroop Color-Word Task again involved adaptive presentation of Stroop stimuli as a function of task performance in similar way to that used in the MATH task. Details about the presentation and a full list of measures obtained during the protocol are provided elsewhere [30].

#### Covariates

Following Koelsch, Enge & Jentschke [43], height, weight, BMI, and age were used as covariates. Height was recorded in units of inches, and weight in pounds. All covariates were treated as continuous variables.

### Procedure

The psychophysiology project was part of a two-day data collection protocol. All measures were conducted in hospital clinics, and the HRV examination was carried out during the morning of the second day of participants’ hospital stay. The two cognitive stressors were presented in randomized order. The total test time was approximately half an hour.

### Analyses

To assess and compare the associations between neuroticism and HRV measures under stress and at baseline we fitted three linear regression models with neuroticism as a dependent variable and a HRV measure at stress, at baseline, and combined as predictors. To assess whether the association between neuroticism and HRV is dependent of diagnosed depression and CVD we next removed participants with either condition from the sample and fit the same regression models as before. To assess direct and indirect associations of neuroticism, HRV and the clinical variables, we fit and compare a series of SEM models. All regression and SEM models are adjusted for age, age^2^ sex, BMI, height and weight. A total of 1,255 subjects were available. Of these, 908 had complete data for variables, including all covariates. Neuroticism was available for 955 subjects. Structural Equation Modelling used Full Information Maximum Likelihood (FIML)[44], and thus utilised all data available. Regressions vary in the effective number of subjects, as reflected in the degrees of freedom in these analyses. The analyses were conducted in the R 3.1.3 environment [45], and SEM models were fitted using the OpenMx package [46].

## Results

Descriptive statistics for the personality and cardiovascular reactivity measures are shown in Table 1. A hundred and forty three (11.4%) participants were classified as having depression, and 144 participants (11.4%) had CVD. Of them, sixteen participants (1.4%) were positive for both depression and CVD. Heart function measures from the Stroop and math challenges correlated highly (*r’s* ranging from .81 to .91, all p-values <.001), and HRV measures from the two challenge conditions were therefore combined in all subsequent analyses. For the subjects who had valid data for one task only (n = 63), the single available measure was used in place of the average.

**Table 1:** Ns, Means and Standard Deviations for Experimental variables and Confounders.

		Female		Male		Total	
	N	M	SD	M	SD	M	SD
Neuroticism	955	2.11	0.63	1.99	0.64	2.05	0.64
Depression	1152	0.91	2.06	0.43	1.42	0.69	1.82
Height (feet)	933	4.99	0.16	5.28	0.45	5.12	0.36
Weight (pounds)	949	161.68	34.83	199.09	36.02	178.59	39.96
BMI	929	27.47	5.95	28.39	4.79	27.88	5.48
SDRR baseline	1152	3.48	0.45	3.54	0.45	3.51	0.45
LF baseline	1152	5.36	1.1	5.61	1.13	4.87	1.26
HF baseline	1152	4.94	1.28	4.78	1.23	5.47	1.12
SDRR stress	1136	3.25	0.45	3.28	0.48	3.26	0.46
LF stress	1136	4.71	1.09	4.88	1.15	4.78	1.12
HF stress	1136	4.56	1.28	4.45	1.24	4.51	1.26

Note: BMI = Body Mass Index, SDRR = Standard Deviation of R to R intervals, LF = Low Frequency Heart Rate Power, HF = High Frequency Heart Rate Power. “Stress” and “baseline” refer to testing under the stress and baseline conditions respectively.

We first used multiple regression to examine whether higher levels of neuroticism were associated with reduced HRV under stress and at baseline. Neuroticism scores were used as the dependent variable and SDRR under stress, and SDRR at baseline as the independent variables, and age, sex, BMI, height, and weight were entered as covariates prior to SDRR. Regression results are described in full in Table 2. The stress model showed a significant overall effect (R^2^ = 0.08, F(8, 903) = 9.43, p <.001), and SDRR had a significant effect in the predicted direction (p <.001). Same was true for the association between neuroticism and SDRR in the baseline condition: the model was again significant (R^2^ = 0.07, F(8, 917) = 9.11, p <.001), and baseline SDRR was significantly associated with neuroticism (p = .002) thus suggesting neuroticism as a trait marker for heart function. We next tested whether SDRR in both baseline and under stress was independently associated with elevated neuroticism. The model containing both variables as predictors again gave a significant overall result (R^2^ = 0.08, F(8, 917) = 9.51, p <.001). However, whichever of the two SDRR variables was entered first rendered the second non-significant, suggesting multicollinearity of these indicators. We therefore computed the mean SDRR under baseline and under stress. A model using this combined SDRR variable as a predictor of neuroticism, using the same covariates as above gave a highly significant effect.

**Table 2:** Relationships between SDRR measure of HRV and Neuroticism.

	Model 1				Model 2				Model 3			
	Est.	S.E.	t	P	Est.	S.E.	T	P	Est.	S.E.	t	P
(Intercept)	3.294	1.907	1.727	.084	3.354	1.896	1.769	.077	3.466	1.894	1.830	.068
Age	-0.015	0.016	-0.959	.338	-0.017	0.016	-1.044	.297	-0.018	0.16	-1.114	.265
Sex (F)	-0.118	0.206	-0.574	.566	-0.105	0.204	-0.514	.608	-0.136	0.204	-0.665	.506
Age^2^	0.000	0.000	-0.026	.979	0.000	0.000	0.064	.949	0.000	0.000	0.103	.918
Age x sex	0.002	0.004	0.508	.612	0.001	0.004	0.356	.722	0.002	0.004	0.524	.601
BMI	0.022	0.031	0.710	.478	0.023	0.030	0.763	.446	0.023	0.030	0.753	.452
Weight	-0.004	0.005	-0.843	.399	-0.004	0.005	-0.876	.381	-0.004	0.005	-0.883	.377
Height	0.004	0.028	0.156	.876	0.004	0.028	0.139	.889	0.004	0.028	0.156	.876
SDRR stress	-0.166	0.048	-3.421	<.001	-	-	-	-	-	-	-	-
SDRR baseline	-	-	-	-	-0.156	0.050	-3.150	.002	-	-	-	-
Mean SDRR	-	-	-	-	-	-	-	-	-0.187	0.052	-3.601	<.001

Note: BMI = Body Mass Index; SDRR = Stand Deviation of R-R Interval; HRV = Heart Rate Variability.

Using a test procedure and covariates identical to those used for SDRR, models treating HF and LF power as predictors of neuroticism showed significant effects, for both baseline and stress conditions, and did not add to the prediction of neuroticism over and above mean SDRR. This ability of both HF and LF power to predict neuroticism has implications for interpretations of HRV measures as indicators of autonomic effects on heart function. It is known that respiratory pattern modulates HRV independent from autonomic regulation [47] and respiratory pattern has been linked to negative affect [48]. While we did not attempt to measure respiration effects, inspiratory vagal control is relatively specific to HF-HRV [49]. This allows us to test, to some extent, the role of respiratory influences relative to autonomic influences. The two power measures were similar in the ability to predict neuroticism (*p*_*HF*_ = .005, *p*_*LF*_ = .003), thus ruling out the possibility that HRV is mostly reflecting respiratory effects on (or of) neuroticism.

We next moved to test our hypothesis that HRV would be related to neuroticism, independent of CVD and depression. To test whether the previous results were dependent on participants in the sample who had been diagnosed with either depression or CVD, we removed these subjects and reran the regression models on this new healthy sample (n = 800). This did not change the sign or the magnitude of any of the associations between HRV measures and neuroticism. Whether recorded under stress or at baseline, SDRR was a significant predictor of neuroticism in the disease free sub-set (p = .001 and.009 respectively). Finally, the combined measure of SDRR under stress and baseline conditions was also significantly related to neuroticism (p = .002). These findings confirmed that the relationships seen between neuroticism and HRV were independent of the presence of CVD and depression.

We next incorporated all our measures into a structural equation modelling framework, to examine simultaneously the relationship among these factors (see Fig 1). As shown in Fig 1, the full model includes direct effects of neuroticism on HRV, on CVD and on depression, and allowed for covariation among HRV and CVD, CVD and depression, and between HRV and depression. The model also included covariates of age, sex, BMI, height, and weight which were allowed to load on neuroticism, HRV, CVD, and depression. For clarity, these covariates are not shown in Fig 1, but all paths and estimates are shown in S1 Table.

Given the indifference of relationships among our variables to the two frequency indicators of HRV, the mean SDRR score was used as the HRV measure in the rest of the analyses. The path between HRV and depression was not significant, and dropping it did not significantly reduce model fit (p = .287). We then moved on to test the association between neuroticism and HRV. Dropping the path from neuroticism to HRV significantly reduced model fit (χ^2^(2) = 15.85, p <.001), supporting association of neuroticism and HRV, independent of other paths.

We next tested the association between neuroticism and depression, with a similar result: Dropping the neuroticism to depression path reduced model fit (χ^2^(2) = 369.23, p <.001). We then tested whether HRV and depression were independently related to CVD. Dropping either the HRV to CVD path, or the path from depression to CVD significantly reduced the model fit (χ^2^(2) = 8.56, p = .014 and χ^2^(2) = 71.47, p <.001, respectively).

Finally, to test the hypothesis that neuroticism has an independent contribution to CVD, we tested dropping the path from neuroticism to CVD—this significantly reduced model fit (χ^2^(2) = 52.02, p <.001) supporting a distinct association of neuroticism on CVD. (See Table 3 for a list of all model comparisons).

**Table 3:** Model comparisons: All paths significant by χ^2^ and AIC comparison except for the HRV with Depression path.

	EP	AIC	Δ -2LL	Δ df	P-value
Model 1 (baseline)	45	0.0027	–	–	–
Drop HRV with Depression	44	-0.8629	1.134	1	.287
Drop path from N to CVD	44	48.941	50.939	1	<.001
Drop path from N to HRV	44	12.718	14.715	1	<.001
Drop path from N to Depression	44	366.103	368.101	1	<.001
Drop covariance of HRV with CVD	44	3.7757	5.773	1	.016
Drop covariance of Depression with CVD	44	66.695	68.692	1	<.001

Note: Model 1 was used as a baseline for all comparisons. N = neuroticism, CVD = cardiovascular disease, HRV = heart rate variability; EP = Estimated Parameters; AIC = Akaike Information Criterion; -2LL = -2× Log likelihood.

## Discussion

We found that neuroticism was significantly associated with HRV. This was true both under stress and at baseline. Structural equation modeling indicated significant associations of neuroticism with HRV, depression, and CVD, with additional covariation between CVD and depression. The direct path between HRV and depression was estimated at near zero and was non-significant, suggesting that the covariation of depression with HRV may not be causal but may rather reflect influences of CVD and neuroticism. Significant associations of neuroticism with depression and CVD, however, suggest that neuroticism is a potential candidate explaining some of the comorbidity of CVD and depression. All the results were robust to the inclusion of covariates of age, sex, height, weight, and BMI. Jointly, then, the results suggest an association of neuroticism with cardiovascular activity, and significant associations with multiple diseases.

The significant association of neuroticism with HRV enhances previous work using different measures of emotionality [50] and of heart function [43,51,52]. Suls and Bunde [10] argued that dispositional negative affectivity may be more important as a causal factor in the development of disease compared to than specific experienced emotion states such as anxiety or hostility—a suggestion corroborated by several studies (e.g. [11]). Our results further support this claim, and suggest that neuroticism, one of the five major domains of personality, may be an excellent measure of the trait affective responsiveness factor influencing heart disease, possibly both directly and via its links to depression and reduced heart reactivity. Such a model would be in keeping with RdOC models of depression [53]. Additionaly, the finding that individual variations in personality dimensions may reflect differences in neurophysiological functioning extends the classic ideas of Eysenck [54] and Gray (for a review of Gray’s BIS and BAS systems in relation to heart function see [55]).

It was interesting that the association between neuroticism and HRV in the baseline condition was comparable to that found for HRV in the stress condition. The finding that the suboptimal cardiac reactivity pattern shown by high-neuroticism individuals was cross-situational is at first glance anomalous: one might expect heart function under stress to more clearly reflect emotional lability. Its presence at baseline may indicate high neuroticism is chronically associated with adverse heart function, not only during maladaptive responses to stress.

### Limitations

This study is not without limitations. HRV under laboratory conditions generalizes only weakly to real-life stress responses [56]. This would act to obscure relationships with HRV reactions to stress. Countering this, in our study, neuroticism was significantly related not only to HRV under stress but also to HRV under baseline conditions, suggesting that cardiovascular function may be related to neuroticism independently of stress. Nevertheless, associations of neuroticism and HRV may be stronger, or show interactions with response to in-vivo stressors and this should be explored. Furthermore, individual differences in HRV may not only reflect individual differences in heart function, but this link may be obscured by their relationship to respiration [47]. However, given that this relationship is more specific to HF measures of HRV [49], and that in our study effects of HF, LF and SDRR measures of HRV all had effects comparable in size and direction, we conclude that the link of HRV to respiration did not affect our results.

Heart disease includes a broad range of conditions with heterogeneous etiologies and presentations. Positive responder to the question about heart disease include patients with conditions ranging from atherosclerotic CHD (the condition most strongly linked with negative affect) to congenital valve effects [29]. Future studies should examine the specificity of links between neuroticism, depression, HRV and specific cardiovascular conditions. Finally, our sample was cross-sectional. Therefore, we were limited in our ability to assign directionality in the associations of HRV and neuroticism. Future studies should include three or more waves of assessment to parse directionality.

In conclusion, our results suggest that HRV is a biomarker for neuroticism, and that neuroticism can account for portions of the phenotypic association of HRV with CVD, and depression. As such, it is a viable target for translational research aimed at reducing risk for these common and serious diseases.

## Supporting Information

S1 Table The full list of paths and estimates for the baseline (saturated) model. Note: Exogenous covariates omitted: standardized covariances equal 1. BMI = body mass index, N = neuroticism, CVD = cardiovascular disease, HRV = heart rate variability.
(DOCX)
