# Supplementary material for: The Association between Neuroticism and Heart Rate Variability Is Not Fully Explained by Cardiovascular Disease and Depression
Source: PLoS One. 2015 May 7;10(5):e0125882. doi: 10.1371/journal.pone.0125882 (PMC4423941; doi:10.1371/journal.pone.0125882)
Supplement: S1 Table — Note: Exogenous covariates omitted: standardized covariances equal 1. BMI = body mass index, N = neuroticism, CVD = cardiovascular disease, HRV = heart rate variability. (DOCX) [file pone.0125882.s001.docx]

*Table S1
The full list of paths and estimates for the baseline (saturated) model.*

| **Path** | **Estimate** | **95% CI** | **df** |
| --- | --- | --- | --- |
| Age to HRV | -0.372 | -0.428, -0.317 | 1152 |
| Age to N | -0.249 | -0.305, -0.192 | 1042 |
| Age to CVD | 0.363 | 0.317, 0.409 | 1249 |
| Age to Depression | 0.025 | -0.022, 0.071 | 1054 |
| Sex to HRV | -0.586 | -0.751, -0.421 | 1152 |
| Sex to N | -0.027 | -0.199, 0.146 | 1042 |
| Sex to CVD | -1.308 | -1.444, -1.172 | 1249 |
| Sex to Depression | 1.133 | 0.996, 1.267 | 1054 |
| BMI to HRV | 0.710 | 0.278, 1.142 | 929 |
| BMI to N | 0.436 | -0.015, 0.886 | 1006 |
| BMI to CVD | 1.498 | 1.412, 1.854 | 1005 |
| BMI to Depression | -0.471 | -0.830, -0.111 | 1010 |
| Weight to HRV | -1.096 | -1.610, -0.592 | 949 |
| Weight to N | -0.529 | -1.055, -0.002 | 1029 |
| Weight to CVD | -1.837 | -2.252, -1.421 | 1028 |
| Weight to Depression | 0.846 | 0.427, 1.266 | 1033 |
| Height to HRV | 0.054 | -0.219, 0.326 | 933 |
| Height to N | 0.138 | -0.147, 0.423 | 1011 |
| Height to CVD | -0.163 | -0.388, 0.062 | 1010 |
| Height to Depression | 0.499 | 0.272, 0.726 | 1015 |
| N to HRV | -0.104 | -0.157, -0.051 | 955 |
| N to CVD | 0.161 | 0.117, 0.204 | 1037 |
| N to Depression | 0.466 | 0.421, 0.510 | 1042 |
| Age with sex | -0.058 | -0.114, -0.002 | 1255 |
| Age with BMI | -0.039 | -0.095, 0.016 | 1010 |
| BMI with sex | -0.091 | -0.147, -0.036 | 1010 |
| Age with weight | -0.060 | -0.116, -0.005 | 1033 |
| Sex with weight | -0.575 | -0.642, -0.514 | 1033 |
| BMI with weight | 0.846 | 0.777, 0.922 | 1010 |
| Age with height | -0.046 | -0.102, 0.009 | 1015 |
| Height with sex | -0.934 | -1.013, -0.862 | 1015 |
| BMI with height | -0.030 | -0.086, 0.026 | 1010 |
| Height with weight | 0.497 | 0.438, 0.562 | 1010 |
| Height with height | 1.000 | 0.926, 1.083 | 1255 |
| HRV with HRV | 0.848 | 0.785, 0.918 | 1255 |
| N with N | 0.927 | 0.858, 1.004 | 1255 |
| CVD with HRV | -0.047 | -0.087, -0.009 | 1147 |
| CVD with CVD | 0.578 | 0.535, 0.625 | 1255 |
| Depression with HRV | 0.021 | -0.018, 0.061 | 966 |
| Depression with CVD | 0.135 | 0.102, 0.169 | 1049 |
| Depression with depression | 0.589 | 0.545, 0.638 | 1255 |

*Note.* Exogenous covariates omitted: standardized covariances equal 1.

BMI = body mass index, N = neuroticism, CVD = cardiovascular disease,

HRV = heart rate variability.
